# Mental Illness and Amyloid: A Scoping Review of Scientific Evidence over the Last 10 Years (2011 to 2021)

**DOI:** 10.3390/brainsci11101352

**Published:** 2021-10-14

**Authors:** Gianluca Pandolfo, Fiammetta Iannuzzo, Giovanni Genovese, Antonio Bruno, Giovanni Pioggia, Sergio Baldari, Sebastiano Gangemi

**Affiliations:** 1 Department of Biomedical and Dental Sciences, Morphological and Functional Images, University of Messina, 98121 Messina, Italy; gpandolfo@unime.it (G.P.); giovannigeno@live.it (G.G.); antonio.bruno@unime.it (A.B.); 2 Institute for Biomedical Research and Innovation (IRIB), National Research Council of Italy (CNR), 98125 Messina, Italy; giovanni.pioggia@cnr.it; 3 Nuclear Medicine Unit, Department of Biomedical and Dental Sciences and of Morpho-Functional Imaging, University of Messina, 98125 Messina, Italy; sergio.baldari@unime.it; 4 School and Operative Unit of Allergy and Clinical Immunology, Department of Clinical and Experimental Medicine, University of Messina, 98125 Messina, Italy; gangemis@unime.it

**Keywords:** APP, amyloid, tau protein, mental illness, Alzheimer’s diseases, schizophrenia, depression, bipolar disorder

## Abstract

Amyloid precursor protein and its derivates represent a central factor in the process of neurodegeneration in Alzheimer’s disease (AD). Since mental illnesses share with AD cognitive impairment, amyloid indicators have been used to explore the unknown pathophysiologic mechanisms underlining psychiatric illness. This work aims to compare the role of amyloid markers, together with tau proteins, among various mental disorders evaluating the possible role of altered amyloid metabolism in the onset and in the course of psychiatric diseases, considering the relationship with cognitive impairment in dementia. This review includes articles written in English, published between 1 January 2011 and 31 January 2021, which evaluated amyloid and tau proteins in psychiatric patients. After screening, 31 studies were included in the review. Results suggest that amyloid metabolism is altered in major psychiatric disorders and that it could be a marker of cognitive impairment. Nevertheless, the role of amyloid in mental diseases seems to be related to neurodevelopmental alteration as well as neurodegeneration processes, like in AD. The role of amyloid in the pathogenesis of mental disorders is still unknown. Amyloid should not be only considered as a marker of cognitive impairment in mental illness, but also for altered neurodevelopment.

## 1. Introduction

The manifestation and course of psychiatric diseases are characterized by great variability. In recent decades, one of the major psychiatric research challenges has been the identification of specific causes of mental illnesses [1]. The difficulty in finding a clear etiopathogenesis of mental disorders led researchers to focus on identifying specific biomarkers, although biological markers of psychiatric patients are never totally distinct from those of healthy controls [2].

Growing evidence indicates that inflammatory pathways are involved in multiple psychiatric disorders, such as schizophrenia (SCZ), bipolar disorder (BD), autism spectrum disorder (ASD), and major depressive disorder (MDD) [3,4,5]. Therefore, research about neuro-immune and neuro-inflammation biomarkers is becoming an increasing field of study, to improve the diagnosis and treatment of mental illnesses [6].

Studies about inflammatory biomarkers related to mental disorders intersect those concerning neurological disorders. Indeed, in neurological disorders, inflammation is a well-known factor and various biomarkers seems to contribute to the development of pathologies in the neurological field [7,8]. Especially in Alzheimer’s diseases (AD), neuroinflammation hypothesis seems to be a significant element to pathogenesis, although links between AD and systemic inflammation is still less unclear [9].

According to previous studies, the amyloid protein may be an important link between inflammation and AD. Indeed, β- amyloid can stimulate microglial activation throughout toll-like receptors (TLRs); this process would be responsible for the secretion of more pro-inflammatory cytokines that actively contribute to neurotoxicity and neuronal dysfunction [10,11].

As a matter of fact, amyloid β-peptide (Aβ) is derived by the altered cleavage process of the amyloid precursor protein (APP), which represents a central factor in the process of neurodegeneration in AD. In addition, even though early studies stressed the influence of amyloid fibrils on neighboring tissues in AD [12], recent studies suggest an importance of soluble forms of precursor proteins [13]. For example, the soluble oligomers of Aβ, rather than insoluble fibrillar Aβ, in the brain correlate with the marker of disease severity in patients with Alzheimer’s disease [14]. Moreover, APP seems to be involved in neurodevelopmental process of the normal brain, since it may play a central role in neural cell migration [15,16].

Psychiatric diseases often present a progressive neurodegenerative process and, for this reason, the assessment of typical cerebrospinal fluid (CSF) or serum biomarkers of AD (tau proteins, Aβ) together with the evaluation of amyloid and tau burden in cortical areas, have been used to explore neurodegenerative indicators to understand the pathophysiologic mechanisms underlying psychiatric illness [17]. For example, several studies have been conducted with the aim of comparing SCZ and dementia or major depressive disorder (MDD) and dementia, considering the hypothesis of the same psychopathological continuum between psychiatric and neurological cognitive impairment [18,19].

Nevertheless, the basis of the principal mental illnesses is still not completely well-known, therefore, amyloid may influence different neurodevelopmental trajectories that lead to different psychiatric disorders, following similar or parallel pathways to those related to dementia.

However, since psychiatric disorders are neurodevelopmental diseases [20] but some of them share cognitive impairment with AD and dementia, we could take into consideration that APP and its derivatives (Aβ), together with tau proteins, would be simultaneously an index of the onset, evolution, and degeneration of mental illnesses, in the same or in a different way compared to dementia.

Thus, the present review aims to compare the presence of amyloid and tau proteins in serum and CSF and the deposition of amyloid and tau in brain areas, among various mental disorders.

The second aim of our review is to evaluate the possible role of altered amyloid metabolism in the onset and course of psychiatric diseases, also taking into account the relationship between cognitive impairment in psychiatric patients, compared with cognitive impairment in dementia.

## 2. Materials and Methods

We searched PubMed and Scopus for papers published between 1 January 2011 and 31 January 2021, with combinations of the following search terms: “Amyloid” AND “Anxiety Disorders” OR “Bipolar and Related Disorders” OR “Mood Disorders” OR “Schizophrenia Spectrum and Other Psychotic Disorders” OR “Disruptive, Impulse Control, and Conduct Disorders” OR “Feeding and Eating Disorders” OR “Neurotic Disorders” OR “Personality Disorders”.

We conducted a preliminary search, which revealed 1201 papers; half of these were concentrated in the last 10 years (631), so we arbitrarily decided to limit the analysis of the papers to the period between 2011 and 2021.

Articles were included in the review according to the following inclusion criteria: written English and containing quantitative and qualitative information on amyloid, tau, and psychiatric disorders. Articles were excluded because they were irrelevant to the topic in question, or reviews, or cases report, or articles about animal models, and genetics or molecular studies.

## 3. Results

**Figure 1:**
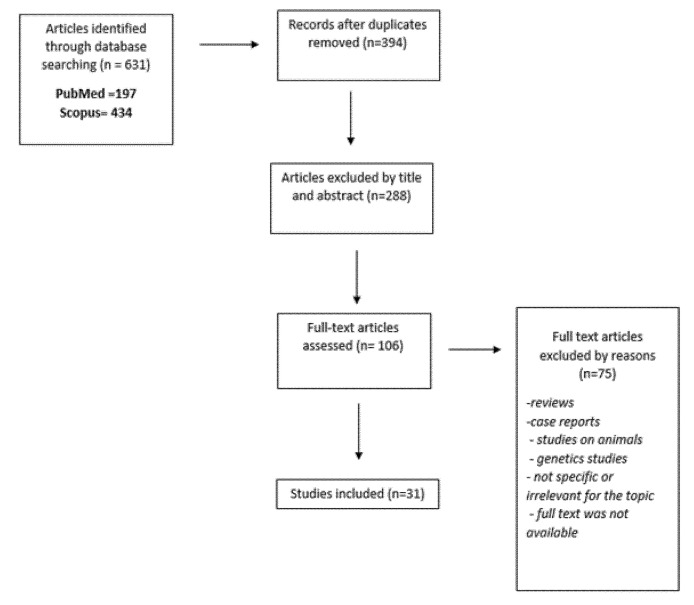
Flow diagram.

We found 631 research papers that evaluated serum amyloid or CSF amyloid in psychiatric patients. After duplicates were removed (*n* = 237) we found 394 articles. Of these, at the first screening, conducted by title and abstract, 288 studies were excluded. After the second screening conducted by full-text examination, 75 articles were excluded because they were reviews, case reports, studies on animals, genetics studies, not specific or irrelevant for the topic, or because the full text was not available. Eventually, 31 studies met the inclusion criteria and were included in the review. The annexed table summarizes the selected articles (Table 1), whereas the annexed flow diagram (Figure 1) summarizes the selection process.

### 3.1. Mental Illness and Amyloid

One study by Hidese et al. [21] described CSF neuroplasticity-associated proteins levels (including amyloid precursor protein-APP) in patients with major psychiatric diseases (schizophrenia, bipolar disorder, and major depressive disorder). They found decreased CSF APP level in patients with schizophrenia or bipolar disorder. One study by Fourier et al. [22] investigated differences in biomarkers in order to discriminate between psychiatric and neurological disease. They evaluated CSF levels of total tau (t-Tau), phosphorylated tau (p-Tau), Aβ42, and neurofilament light chain protein (NfL) in patients with psychiatric diseases (depressive disorders, anxiety disorders, bipolar disorders, schizophrenia, and post-traumatic disorders) compared with patients with neurodegenerative disorders (probable AD, behavioural variant of frontotemporal dementia, Creutzfeldt–Jakob disease, Lewy Body disease, and probable progressive supranuclear palsy). The result was that t-Tau and NfL exhibited better diagnostic performances than p-Tau and Aβ42 to discriminate between the two samples. Increased t-Tau, p-Tau, and NfL and decreased Aβ42 were observed in patients with neurodegeneration compared to psychiatric patients.

### 3.2. Schizophrenia

Three studies [23,24,25] analysed β-amyloid and tau protein in CSF of elderly patients with schizophrenia, compared with a biomarker profile of Alzheimer diseases.

In Frisoni et al. [23], CSF tau concentration in older schizophrenia patients was within normal limits, while CSF Aβ42 levels were significantly lower compared to healthy elders, but higher than in AD patients.

Research of Albertini et al. [24] found that in CSF from schizophrenia patients, there was a strong reduction of almost all Aβ species while in AD, Aβ42 was the only peptide reduced.

A study by Seppala et al. [25] compared older patients with recent psychotic symptoms and subjects with chronic schizophrenia. Only one patient out of 25 with a psychiatric diagnosis and none out of the comparison group had a CSF profile typical of AD.

One case–control study by Tereshkina et al. [26] evaluated APP in peripherical blood of 24 schizophrenic patients, compared with healthy controls. The result was that intensity APP (molecular masses of 130 kDa) and APP ratio were significantly reduced in schizophrenia patients compared to the controls.

### 3.3. Depression

Six studies described the relationship between depression and tau and amyloid biomarkers in CSF (Aβ40, Aβ42, t-tau, and p-tau).

In three of these studies [27,28,29], the response to a treatment was considered. Clarke et al. [27] observed an alteration in Aβ40 and p-tau in patients with major depressive disorders (MDD) receiving antipsychotic drugs, compared with those not receiving them. Kranaster et al. [28] observed a correlation between the response to electroconvulsive therapy (ECT) and the increment of Aβ42, whereas Kranaster et al. [29] explored the involvement of CSF levels of p-tau, t-tau, and Aβ40 in reduction of depressive symptoms after ECT.

Two of these studies [30,31] analysed a correlation between MDD, CSF levels of amyloid biomarkers, and AD. The case–control study by Pomara et al. [27] observed reductions in CSF levels of Aβ42 in a patient with MDD, similar to what happens in AD. However, the case–control study by Reis et al. [28] found a difference in Aβ42 levels and T-tau levels in AD patients compared to MDD.

Finally, Pomara et al. [32] measured, in 28 patients with late-life major depression (LLMD) and 19 healthy controls, CSF levels of Aβ42, Aβ40, t-tau and p-tau at baseline and at the three-year follow-up visit. The authors found an increment in CSF Aβ42 associated with more severe symptoms, and an increment of Aβ42, Aβ40, and t-tau not associated with cognitive decline.

Seven studies analysed the correlation between depression and levels of amyloid and tau in serum.

Three of these studies [33,34,35] found that serum Aβ40/Aβ42 ratio was significantly higher in MDD patients, compared with the controls. Inoue et al. [35] found a higher Aβ40/Aβ42 ratio in both older and younger patients; Namekawa et al. [34] found a higher Aβ40/Aβ42 ratio in elderly patients with early or late onset of MDD.

Yasuda et al. [36] showed a correlation between serum Aβ42 levels and Aβ oligomers in MDD patients compared with controls. Direk et al. [37] noted that a correlation between high plasma Aβ levels and significant depressive symptoms in the elderly was related to prodromal dementia, whereas the relationship between low plasma Aβ levels and depressive symptoms was not related by dementia.

Yamazaki et al. [38] found that depression and AD belong to the same continuum.

The clinical trial by Zimmermann et al. [39] explored plasma concentrations of β-amyloid peptides in 13 patients with depressive episodes, before and after electro-convulsive therapy (ECT), highlighting an increment in plasma concentrations of all peptides within 30 min after the ECT.

Five studies concerned the deposition of tau and β-amyloid in brain regions, investigated by PET radioligands.

One of these studies by Moriguchi et al. [40] explored tau and β-amyloid PET ligand retention in 20 elderly patients with MDD and 19 controls. Whereas four of these studies [41,42,43,44] found increment in radioligand binding for β-amyloid, in various brain regions, in patients with MDD, compared with the controls.

In a cross-section neuroimaging study by Kumar et al. [44] [(18)F]FDDNP binding was significantly higher in the posterior cingulate and lateral temporal regions.

In a case–control study by Wu et al. [43], increased 18F-florbetapir binding was found in the parietal and precuneus cortex.

In a study published in February 2018 by Wu et al. [41], increment of ^18^F-florbetapir binding (in specific cortex areas such as the precuneus, parietal, and posterior cingulate cortex) was related to a decreased plasma Aβ42 level, a lower Aβ42/Aβ40 ratio, and an increased plasma Aβ40 level.

In a study published in May 2018 by Wu et al. [42], amyloid binding was compared between MDD patients with and without mild cognitive impairment (MCI). The main finding was that amyloid deposition was higher in MCI–MDD patients, intermediate in not MCI–MDD patients, and lower in controls.

### 3.4. Bipolar Disorder

Three studies analysed correlations between bipolar disorder (BD) and amyloid and tau levels in CSF.

Jakobsonn et al. [45] found that amyloid precursor protein (APP) metabolism was altered in bipolar disorder. Rolstand et al. [46] showed increased Aβ42/40 ratios associated with cognitive impairment. Forlenza et al. [47] studied Aβ42, T-tau, and P-tau in CSF of bipolar patients with cognitive impairment, showing a different pattern of CSF amyloid in BD, compared to Alzheimer’s disease.

Two studies conducted by Piccinni et al. [48,49] regarded bipolar depression and amyloid and tau biomarkers in serum, in this case analysing the relationship with cognitive impairment.

In both studies, high levels of Aβ40/Aβ42 ratio were correlated with the risk of cognitive impairment.

### 3.5. Postmortem Studies

Two studies [50,51] analysed the postmortem brain of depressed patients. A study by Wilson et al. [50] did not find a relationship between major depression and neurodegenerative process analysing dementia markers such as tau tangles, beta-amyloid plaques, and Lewy bodies. A study by Saldanha et al. [51] showed that amyloid plaque density was associated with clinical dementia but not with depression.

## 4. Discussion

To our knowledge, this is the first review to explore the role of β-amyloid and tau proteins in notable mental illnesses. Over the last 10 years, many studies have explored the role of amyloid in mental illness, both as a biomarker and a pathogenic element.

A study about CSF APP levels in patients with schizophrenia or bipolar disorder by Hidense and collaborators [21] suggested that neuroplasticity-associated proteins may be used as markers in psychiatric disorders. Research by Fourier et al. [22] on heterogeneous samples of psychiatric patients evaluated the usefulness of amyloid and tau protein as markers of differential diagnosis between psychiatric and neurodegenerative diseases, since the distinction between psychiatric and neurodegenerative disorders is often a challenge in daily practice.

Other studies explored the relationship between neurodegeneration in Alzheimer’s disease and neurodegeneration in schizophrenia, since patterns of cognitive impairment, similar to dementia, were observed in elderly patients with schizophrenia. According to these researchers [23,25], schizophrenia patients did not show a profile of CSF biomarkers typical for Alzheimer disease. In particular, the work by Frisoni et al. [23] concluded that older schizophrenia patients display a peculiar pattern of CSF amyloid and tau biomarkers, mostly related to a neurodevelopmental effect, and not to a neurodegeneration process. Even so, Albertini et al. [24], as well as Tereshkina et al. [26] demonstrated the presence of altered APP metabolism in schizophrenia.

Since plasma levels of beta amyloid have been linked to dementia, and depression seems to precede the onset of dementia, several studies had investigated a possible evolution from depression to dementia mediated by amyloid. These researches led to conflicting results. For example, whereas Yasuda et al. [36] suggested a possible evolution from depression to AD and Yamazaki et al. [38] found that depression and AD belong to the same continuum, Direk et al. [37] suggested that amyloid peptides had a role in depression etiology, different than in dementia.

However, most of the analysed studies proposed an altered amyloid metabolism in depression [30,31,32].

In addition, conflicting results emerged in the assessment of amyloid protein in CSF of patients with depression. Indeed, if studies such as Pomara et al. [30] proposed reductions in CSF levels of amyloid in patients with MDD, similar to what happens in AD, studies such as Reis et al. [31] found a difference in amyloid and tau levels in AD patients compared to MDD, suggesting a different role of amyloid metabolism in depression, compared with dementia. A 2012 study by Pomara et al. [32] speculated that reduction in CSF levels of Aβ42 might be related to increased brain amyloid beta plaques or decreased soluble amyloid beta production in elderly individuals with major depression.

According to PET studies deposition, tau deposition might be related to cognitive decline in patients with depression [41,42], but some results also indicated that tau deposition might play a role in the pathophysiology of MDD, suggesting tau deposition as a signal of neuronal dysfunction [40].

Post-mortem studies in depressed patients [50,51] did not find a relationship between major depression and dementia, suggesting that the link between dementia and depression could not be mediated by β-amyloid.

The investigation about CSF amyloid and tau levels in bipolar patients showed altered APP metabolism also in bipolar disorder [45]. Cognitive impairment in BD has been interpreted as a response to neurotoxicity in a study exploring CSF biomarkers of neurodegeneration in bipolar patients [46], and, even so, bipolar disorder seemed not to display a typical CSF pattern of Alzheimer’s disease [47].

Instead, in studies concerning bipolar depression, amyloid seemed to be a biomarker of cognitive impairment.

Notably, studies on schizophrenia and bipolar disorder seem to agree on the altered metabolism of APP, probably connected to an unknown neurodevelopmental process. In schizophrenia, genetic alterations, i.e., in BACE1, could play a key role [23,52]. In bipolar disorder, alterations in amyloid could be linked to neurotoxic factors [46]. In both schizophrenia and bipolar disorder, amyloid and tau are indicators of cognitive impairment.

However, studies about bipolarism did never seem to concern the maniacal phase of the disease, investigating either the disorder as a whole or the depressive phases related to cognitive impairment.

Nevertheless, most of the evaluated research concern depression, and the relationship between depression and cognitive decline in dementia. These studies sometimes agree on a neurodegenerative altered metabolism similar to that of dementia, sometimes indicate an alteration in depression that follows different pathways, compared to AD.

However, a common element between various major psychiatric diseases appears to be the altered metabolism of APP, and the role of amyloid and tau as a biomarker of cognitive impairment. In contrast, the differences in pathogenetic processes involving amyloid among the various diseases are confusing.

Thus, according to the shown data, the role of β-amyloid and tau proteins in the genesis and evolution of mental illness is still unclear.

Both serum and CSF levels of amyloid and amyloid beta plagues seem to differently influence the pathogenesis of mental disorders. Further studies are needed to investigate the correlation between these biomarkers, in particular between protein level and protein aggregation level.

Researchers have observed that amyloid metabolism may be altered both in affective disorders and in the schizophrenic spectrum [26,53] but most of the studies examined the relationship between mental disorders and cognitive impairment, and few have assessed the genetic and molecular aspects underlying the mechanisms of altered amyloid metabolism in mental disorders.

Indeed, psychiatric diseases are neurodevelopmental disorders, although they lead to neurodegenerative phenomena [54]. Even though the role of altered APP metabolism is clear in Alzheimer’s disease, little is known about its role in the brain during neurodevelopment. For example, APP seems to be involved in the correct migration of neuronal precursor cells to the cortical plate and in neural network formation [55], and these observations suggest that APP’s role in neurodevelopmental diseases must be explored further.

**Figure 2:**
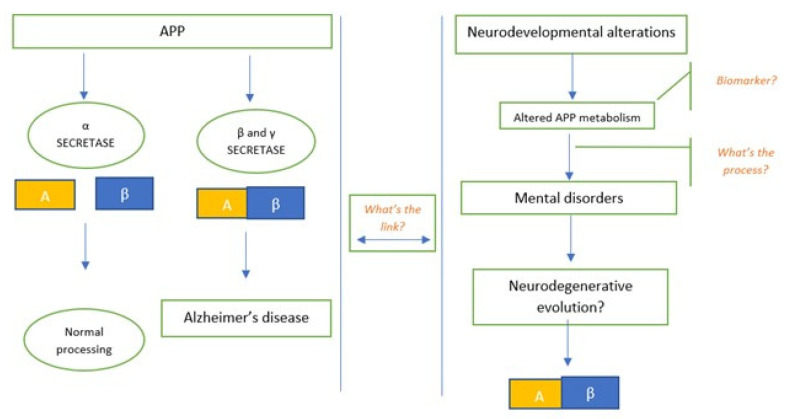
Altered APP pathways in mental disorders could intersect with the neurodegeneration processes of dementia, without sharing the same trajectories. The figure explains the need for a link between the neurodegenerative evolution of APP in the genesis of AD, and the still unknown process behind mental disorders.

Moreover, the analysed studies show that the role of amyloid in the degeneration of mental disorders follows different pathways, compared to the typical degeneration of dementia. This observation suggests that APP, its isoforms, and peptides (β-amyloid) should not be only regarded as markers of neurodegeneration in mental illness, but also for altered neurodevelopment. Indeed, altered APP pathways could intersect with the neurodegeneration processes of dementia, without sharing the same trajectories (Figure 2).

One of the limits of the analysed studies is that the elderly population was examined, suggesting that further longitudinal studies are probably necessary to investigate APP metabolism in mental illness—from youth to old age.

Studies analysing amyloid in response to therapeutic effects (of drugs or electroconvulsive therapies) further suggest the role of amyloid not only as a biomarker but also as a pathogenic element [27,28,29,39]; increment of Aβ peptides in plasma after electroconvulsive therapy suggests the possible role of amyloid as a marker of response to therapy. In particular, in a study by Kranaster and collaborators [29], tau protein, its phosphorylated isoform, Aβ40, and neurogranin were correlated with any form of therapeutic effect. Another study by Kranaster et al. [28] observed a specific antidepressant mechanism not based on a general increase of Aβ, but on the specifically increment of Aβ42, the isoform with the highest amyloidogenic potential. A study by Clarke et al. [27] showed that antipsychotic drugs used in depressed patients may be associated with alteration of Aβ40 and total tau; this data suggests a strong link between depression and progressive organic brain disease. Zimmermann et al. [39] observed an increment of Aβ peptides shortly after ECT session and normalization of their value after two hours.

In conclusion, according to the latest evidence, we can speculate that amyloid and its derivates should not be only considered as markers of cognitive impairment in mental illness, but also for other processes, possibly related to altered neurodevelopment or neuroinfammation. In addition, we can suppose that there probably is a link between the processes of neurodegeneration in mental illness and neurodegeneration in dementia. In addition, further investigation into amyloid as a marker of response to therapies, and as a possible therapeutic target, applied to mental disorders, are required, since they seem to not have been examined.

**Figure 3:**
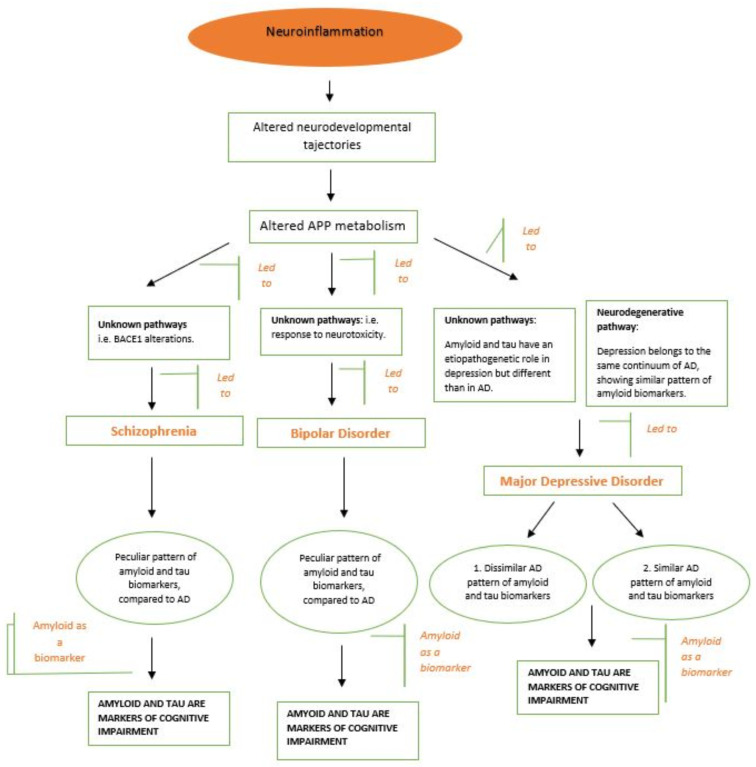
Hypothetical role of APP in the pathogenesis of mental illness, with differences among three of the major mental disorders. The process of neuroinflammation, which led to an altered APP metabolism, might be made up of different pathways for each mental pathology. Thus, whereas in schizophrenia, the pathway could be an alteration in BACE1 and in bipolar disorder it could be a response to neurotoxicity, depression might be influenced by two distinct ways—a neurodegenerative pathway (similar to AD) and an unknown pathway (different from AD).

However, caution is needed in the interpretation of these data. If we want to assume a role of APP in the pathogenesis of mental illness (Figure 3), longitudinal studies are necessary to investigate psychiatric samples from youth to old age. Also, molecular and genetic research should be conducted to clarify the role of APP in the processes of neurodevelopmental alteration and to understand if processes leading to neurodegeneration in mental disease are different from those observed in dementia.

## Figures and Tables

**Table 1:** Summary of the selected articles.

Reference	Type of Study	Subjects	Assessment	Main Findings
Hidese et al., 2020	Case–control study	94 patients with schizophrenia (SCZ), 68 with bipolar disorder (BD), 104 with major depressive disorder (MDD), and 118 healthy controls.	Assessed amyloid precursor protein (APP), BDNF, and other biomarkers.	Decreased CSF APP level in patients with schizophrenia and in those with BD.
Fourier et al., 2020	Case–control study	64 primary psychiatric patients and 162 patients with neurodegenerative disorders.	t-Tau, p-Tau, Ab42 peptide and neurofilament light chain protein were analysed in CSF.	The combination t-Tau + NfL is an association marker that permits differential diagnosis between psychiatric diseases and neurodegeneration.
Tereshkina et al., 2020	Case–control study	24 subjects with a diagnosis of acute paranoid schizophrenia (DSM-IV) and 20 controls.	The study analysed changes in amyloid precursor protein (APP).	Altered APP metabolism in schizophrenia.
Seppala et al., 2014	Prospective case–control study.	51 patients with recent psychotic symptoms and 12 comparison subjects with chronic schizophrenia over 10 years.	Levels of CSF Ab42, tau and p-tau-181 measured by ELISA.	Psychotic patients who had suffered schizophrenia for decades did not display a profile of CSF biomarkers typical for Alzheimer disease.
Albertini et al., 2012	Case–control study	20 subjects with AD, 11 elderly SCZ patients and 20 cognitively healthy controls.	Levels of CSF Ab40, Ab42, and total and P-tau proteins were determined by ELISA.	Altered APP metabolism in schizophrenia.
Frisoni et al., 2011	Case–control study.	11 older schizophrenia patients, 20 AD patients and 6 elderly controls.	CSF biomarkers of brain amyloidosis (Abeta42) and neurodegeneration (total and p-tau).	Older schizophrenia patients show a peculiar pattern of CSF Abeta42 and tau concentrations not consistent with neurodegeneration.
Moriguchi et al., 2020	Case–control study	20 patients with major depressive disorder (MDD) and 19 healthy controls.	Patients and controls were examined by PET with a tau radioligand, [^11^C]PBB3, and an Aβ radioligand, [^11^C]PiB.	Tau depositions may underlie MDD, especially in patients with psychotic symptoms.
Yasuda et al., 2020	Case–control study	104 patients with MDD and 138 healthy participants.	Serum levels of Aβ40, Aβ42 and Aβ oligomers were evaluated.	The MDD group had higher Aβ40 and lower Aβ42 serum levels compared with the control group. Possible evolution from depression to dementia mediated by A β42.
Saldanha et al., 2020	Cross-sectional study	1013 deceased subjects submitted to autopsy, with lifetime history of MDD.	Immunohistochemistry with antibody against β was performed in selected areas.	Neuritic plaque density was associated with clinical dementia but not associated with lifetime or late-life depression (LLD).
Kranaster et al., 2019	Clinical trial study	12 patients with treatment resistant depressive episode, submitted to electroconvulsive therapy (ECT).	Before the first ECT session and between one and seven days after the last ECT session, all CSF samples were drawn.	Tau protein, Aβ1-40 and neurogranin, were correlated with response to ECT.
Wu et al., 2018	Case–control study	63 elderly patients with major depressive disorder (MDD), subdivided into those with mild cognitive impairment (MCI) (*n* = 24) and non-MCI (*n* = 39) patients, and 22 control subjects.	^18^F-florbetapir positron emission tomography imaging used as a biomarker of cerebral amyloidosis and the hippocampal volume used as a biomarker for neurodegeneration.	MCI-MDD patients had significantly higher amyloid deposition and greater hippocampal atrophy, followed by non-MCI MDD patients, as compared to the control subjects.
Wu et al., 2018	Clinical trial	36 non demented MDD patients.	PET imaging to evaluate amyloid brain deposition and blood sample at the same time to measure the plasma levels of Aβ40 and Aβ42.	Decreased plasma Aβ42 level and a lower Aβ42/Aβ40 ratio, in addition to an increased plasma Aβ40 level, were found to be associated with increased ^18^F-florbetapir binding in specific cortex areas.
Yamazaki et al., 2017	Clinical trial	42 patients with depressive episode.	Plasma amyloid levels.	LLD of BD and AD represent a possible clinical continuum and plasma A40 may have a significant role as a predictive biomarker.
Pomara et al., 2016	Clinical trial	47 patients: 28 with LLMD and 19 healthy controls.	CSF levels of Aβ42, Aβ40, t-tau and p-tau were measured at baseline and at the 3-year follow-up visit.	State-dependent association between CSF Aβ42 and depressive symptoms.
Kranaster et al., 2016	Clinical trial	12 patients with MDD.	CSF samples were drawn before the first ECT session and between one and seven days after the last ECT session.	Increase of Aβ1-42 after ECT was found in all patients with clinical response to the treatment, but not in those who did not respond.
Inoue et al., 2016	Case–control study	70 cognitively intact patients with MDD and 81 healthy participants.	Serum Aβ40 and Aβ42 levels were measured. Serum levels of albumin-Aβ complexes (SLAAC) were measured.	SLAAC is decreased in elderly patients with major depression. This decrease was not seen in younger patients. The serum-free Aβ40/Aβ42 ratio was higher in patients with depression even in younger patients.
Forlenza et al., 2016	Case–control study	72 older adults: patients with BD and mild cognitive impairment (BDMCI) (*n* = 16), patients with dementia due to AD (*n* = 17), patients with amnestic MCI (aMCI; *n* = 14), and cognitively healthy older adults (control group; *n* = 25).	CSF concentrations of Ab1-42, T-tau and P < -tau were determined.	Cognitively impaired patients with BD do not display the so-called AD bio-signature in the CSF.
Wilson et al., 2016	Longitudinal clinical-pathologic study	1965 older participants.	Assess beta-amyloid plaques and tau-tangles in 8 brain regions post-mortem.	Results do not support the hypothesis that major depression is related to neurodegenerative or cerebrovascular conditions underlying late-life dementia.
Wu et al., 2014	Case–control study	25 depressed patients and 11 nondepressed comparison subjects who did not meet the diagnostic criteria for AD or mild cognitive impairment.	18F-florbetapir used for amyloid PET data acquisition.	Increased 18F-florbetapir uptake in specific brain regions in patients with late-life depression relative to comparison subjects.
Direk et al., 2013	Longitudinal population-based cohort study	980 participants evaluated for depressive symptoms.	Researchers’ longitudinal association between Aβ levels and depressive symptoms after excluding participants with dementia during follow-up.	Cross-sectional association between high plasma Aβ levels and clinically relevant depressive symptoms in the elderly is due to prodromal dementia. Aβ peptides may play a distinct role on depression etiology.
Namekawa et al., 2013	Case–control study	89 inpatients with MDD divided into two groups based on age at onset of MDD: <60 years and ≥60 years, 111 healthy controls.	Evaluated serum Aβ40 and Aβ42 levels, the Aβ40/Aβ42 ratio.	The serum Aβ40/Aβ42 ratio was significantly higher in elderly patients with both early-onset and late-onset MDD than in age-matched controls.
Pomara et al., 2012	Case–control study	28 cognitively intact patients with major depression and 19 healthy controls.	Analysed CSF levels of amyloid beta 40 and 42.	Elderly, cognitively intact individuals with major depressive disorder have reductions in CSF levels of amyloid beta 42 similar to individuals with Alzheimer’s disease or mild cognitive impairment.
Reis et al., 2012	Case–control study	52 with a diagnosis of MDD, AD, and healthy controls.	Measurement of CSF P-tau_181_, T-tau, and Aβ42 was performed using commercial assays (ELISA).	CSF Aβ42 levels were significantly lower and T--tau levels were significantly higher in AD patients as compared to MDD and control groups. Aβ42/T--tau and Aβ42/P--tau ratios were significantly lower in AD patients as compared to the MDD and healthy groups.
Baba et al., 2012	Case–control study	93 patients with major depressive disorder (MDD) and 413 healthy controls.	Serum Aβ40 and Aβ42 levels, Aβ40/Aβ42 ratio, and other clinical and biological factors were compared between controls.	Serum Aβ40/Aβ42 ratio was significantly higher in MDD patients than in the controls.
Kumar et al., 2011	A cross-section neuroimaging study	20 patients with MDD and 19 healthy control individuals.	[(18)F]FDDNP binding (PET measurement for amyloid and tau) in critical brain regions.	[(18)F]FDDNP binding was significantly higher overall and in the posterior cingulate and lateral temporal regions in the MDD group, compared to the controls.
Zimmermann et al., 2012	Clinical trial	13 patients.	Plasma concentrations of amyloid b (Ab) peptides before ECT, within 30 min after, and 24 h after ECT treatment.	Increase of the plasma concentrations of all four peptides within 30 min after the ECT, followed by the normalization of the peptides concentrations 2 h after the ECT.
Clarke et al., 2011	Cross-sectional study	32 patients with resistant MDD divided into two groups according to treatment with antipsychotics.	CSF tau and amyloid concentrations.	Increments of Ab 1–40 and total levels of tau in V-CSF, in patients with treatment-resistant MDD receiving antipsychotics compared to those individuals not receiving antipsychotic treatment.
Rolstand et al., 2015	Case–control study	82 euthymic bipolar disorder patients and 71 healthy controls.	CSF concentrations of total and phosphorylated tau, amyloid beta (Aβ)1-42, ratios of Aβ42/40 and Aβ42/38, soluble amyloid precursor protein α and β, and neurofilament light chain protein.	CSF biomarkers of neurodegeneration were associated with cognitive performance in euthymic bipolar disorder, but not in healthy controls.
Piccinni et al., 2013	Clinical trial	25 patients suffering from bipolar I or II depressive episodes with or without psychotic symptoms according to DSM-IV-TR criteria.	Aβ40 and Aβ42 were measured by ELISA assay in patients before (T0) and 1 week after (T1) the end of ECT.	Low Aβ40/Aβ42 ratio might characterize a subgroup of depressed patients who respond to ECT, while higher values of this parameter seem to be typical of more severe cases of patients with cognitive impairment.
Jakobsson et al., 2013	Randomized controlled trial	139 bipolar patients and 71 healthy controls.	Neuropsychological assessments+ Analysis of the CSF concentrations of sAPP-a and sAPP-b, and Ab38, Ab40, and Ab42, hyperphosphorylated-Tau (P-tau), totaltau (T-tau), and Ab1-42.	Amyloid precursor protein metabolism is altered in bipolar disorder.
Piccinni et al., 2012	Case–control study	16 patients with bipolar depression type I or II and 16 control subjects.	Levels of Aβ40 and Aβ42 were measured by using specific ELISA kits.	Patients presented significantly lower plasma Aβ42 levels and higher Aβ40/Aβ42 ratio, as compared with control subjects. Positive correlation between the Aβ40/Aβ42 ratio and the number of affective episodes.

## Data Availability

https://pubmed.ncbi.nlm.nih.gov/; info.scopus.com.
